# Supplementary material for: Dynamic-quenching of a single-photon avalanche photodetector using an adaptive resistive switch
Source: Nat Commun. 2022 Mar 21;13:1517. doi: 10.1038/s41467-022-29195-7 (PMC8938474; doi:10.1038/s41467-022-29195-7)
Supplement: Supplementary file 1 — Supplementary information [file 41467_2022_29195_MOESM1_ESM.pdf]

Supplementary Information

for

Dynamic-quenching of a single-photon avalanche photodetector using an adaptive resistive switch

Authors: Jiyuan Zheng[1,2]*, Xingjun Xue[3], Cheng Ji[1], Yuan Yuan[3], Keye Sun[3], Daniel Rosenmann[4], Lai Wang[2,5], Jiamin Wu[2,6], Joe C. Campbell[3], Supratik Guha[1,7]

Affiliations:

[1] Pritzker School of Molecular Engineering, the University of Chicago, Chicago, IL 60637 USA.

[2] Beijing National Research Center for Information Science and Technology, Tsinghua University, Beijing 100084, China.

[3] Electrical and Computer Engineering Department, University of Virginia, Charlottesville, Virginia 22904, USA

[4] Center for Nanoscale Materials, Argonne National Laboratory, Argonne, IL 60439, USA.

[5] Department of Electronic Engineering, Tsinghua University, Beijing 100084, China.

[6] Department of Automation, Tsinghua University, Beijing 100084, China.

[7] Material Science Division, Argonne National Laboratory, Argonne, IL 60439, USA.

*Please address correspondence and requests for materials to J. Z. (email: zhengjiyuan@mail.tsinghua.edu.cn);

Table of Contents for Supplementary Information

The ARS profile **3**

The calculation for Photon Counting with Oscilloscope data **3**

Counting rate 3

Jitter 3

A discussion about simulation discrepancies **5**

Supplementary References **6**

The ARS profile

a b

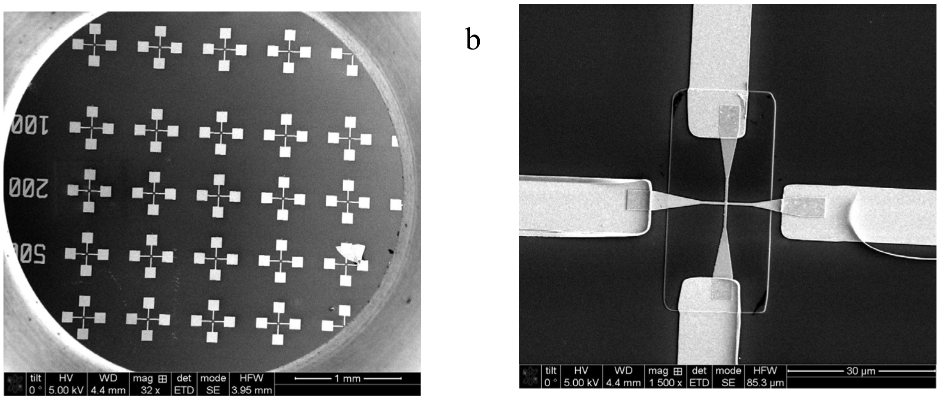

Figure s1. Electron microscope photographs of the MFRS profile. (a) Low magnification image showing 25 independent devices on a wafer. (b) High magnification image showing the cross-bar architecture.

The calculation for Photon Counting with Oscilloscope data

Counting rate

A threshold is set to get the effective counting from the avalanche response curve saved from the oscilloscope. The counting principle is shown in Fig. s2. A timer controls the counting. When the laser pulse comes, the timer is triggered. When the timer is running and the avalanche pulse exceeds the threshold, the timer is stopped, and the pulse is regarded as an effective counting. Then, the timer is reset only when the next laser pulse comes. This counting mechanism is widely used in commercial photon-counting technology[1].

Jitter

Due to the randomness of avalanche, each avalanche pulse has different timing performance, the current pulse trigger the counter at different time pace. A program has been edited to collect the timing histogram of counting and thereafter the jitter as shown in Fig. 2c can be plotted.

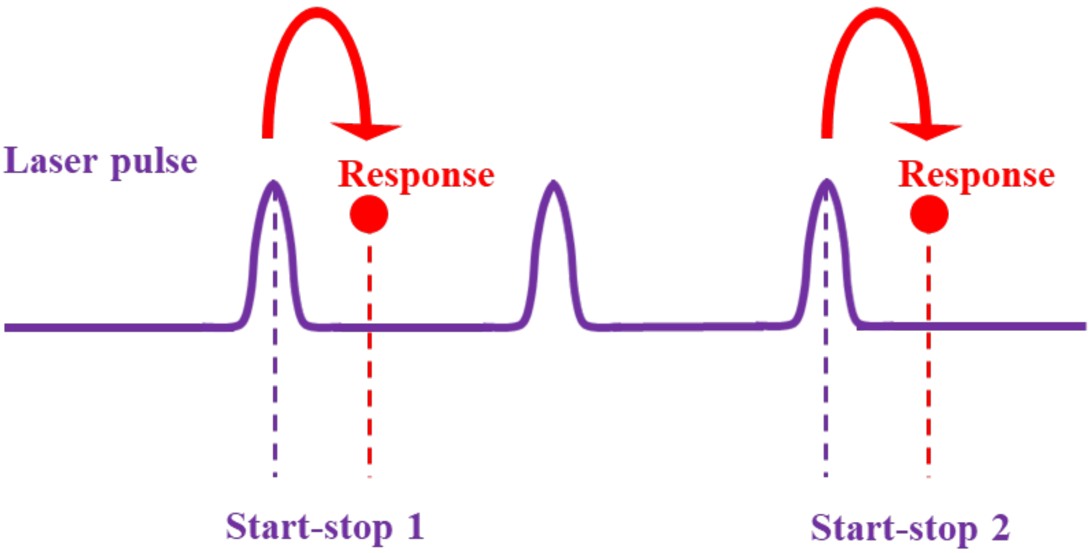

Figure s2. Principle of counting. Counting timer starts with the laser pulse arrival and stops with the response exceeding threshold.

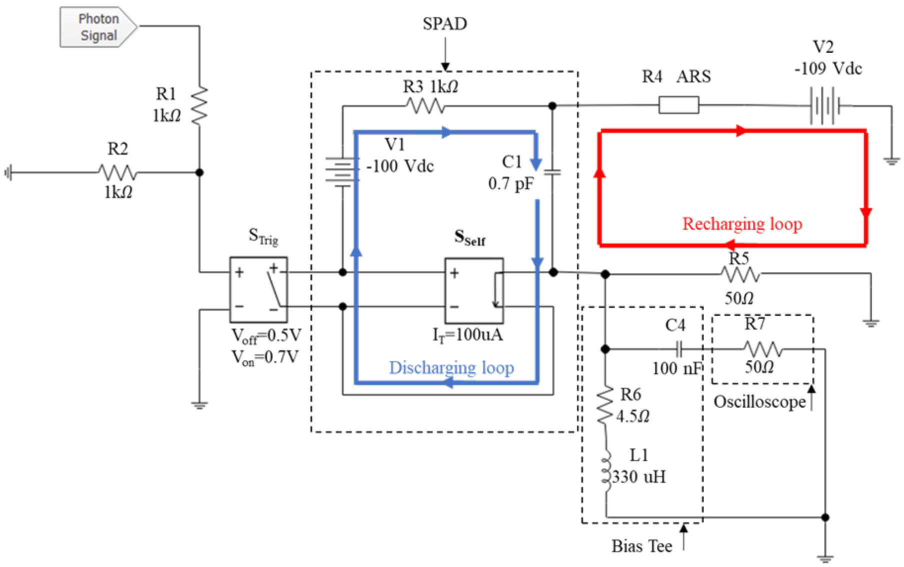

Figure s3. Schematic circuit diagram for Pspice simulation. The photon signal port, *R1*, *R2*, S_{Trig}, S_{Self} are used to mimic the optical switch in Fig. 2. *V1* and *R3* are the equivalent internal voltage source (breakdown voltage) and SPAD's internal resistance, respectively. *C1* is the SPAD junction capacitance. The equivalent optical switch, V1, R3 and C1 form the equivalent circuit of SPAD. *R4* denotes the quenching resistance (ARS). *V2* is the external voltage source. R5 is the 50 Ω impedance matching resistor. C4, R6, and L1 form a Bias Tee, which separates the AC signal and the DC signal. The AC signal is introduced from C4 into an

oscilloscope (R7), of which the input impedance is 50 Ω. The discharging path (blue) and recharging path (red) are labelled. The current flowing through R7, the voltage across SPAD (C1), and the voltage and current on ARS are monitored in simulation.

A discussion about simulation discrepancies

In the Pspice simulation, a hypothesis has been made for the ARS that the resistance switches between fixed off-state value and on-state value. The transition is linear, while the resistance is limited to off-state value or on-state value if its value be beyond the transition window. The hypothesis fits well when the time scale is short (Fig. 4d), however, it fails to fit I-V curve (Fig. s4). The I-V sweep has a longer time scale and a more complex nonlinear model without limit should be used to have a better fitting.

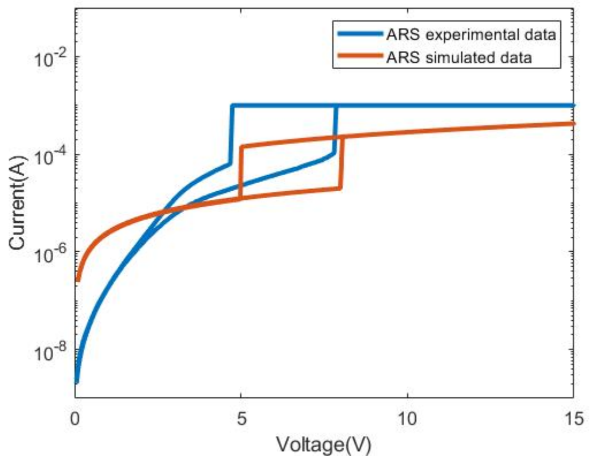

Figure s4. Discrepancies in I-V simulation by using simple linear model to describe the complex dynamic switching of the ARS

Supplementary References

1 Goedhart, J. *et al.* Structure-guided evolution of cyan fluorescent proteins towards a quantum yield of 93%. *Nat. Commun.* **3**, doi:10.1038/ncomms1738 (2012).
